# Structural and biochemical characterization of the environmental MBLs MYO-1, ECV-1 and SHD-1

**DOI:** 10.1093/jac/dkaa175

**Published:** 2020-05-28

**Authors:** Christopher Fröhlich, Vidar Sørum, Sandra Huber, Ørjan Samuelsen, Fanny Berglund, Erik Kristiansson, Stathis D Kotsakis, Nachiket P Marathe, D G Joakim Larsson, Hanna-Kirsti S Leiros

**Affiliations:** d1 The Norwegian Structural Biology Centre (NorStruct), Department of Chemistry, UiT The Arctic University of Norway, Tromsø, Norway; d2 Department of Pharmacy, UiT The Arctic University of Norway, Tromsø, Norway; d3 Department of Laboratory Medicine, University Hospital of North Norway, Tromsø, Norway; d4 Norwegian National Advisory Unit on Detection of Antimicrobial Resistance, Department of Microbiology and Infection Control, University Hospital of North Norway, Tromsø, Norway; d5 Department of Mathematical Sciences, Chalmers University of Technology, Gothenburg, Sweden; d6 Department of Infectious Diseases, Institute of Biomedicine, The Sahlgrenska Academy, University of Gothenburg, Gothenburg, Sweden; d7 Centre for Antibiotic Resistance Research (CARe) at University of Gothenburg, Gothenburg, Sweden; d8 Institute of Marine Research, Bergen, Norway

## Abstract

**Background:**

MBLs form a large and heterogeneous group of bacterial enzymes conferring resistance to β-lactam antibiotics, including carbapenems. A large environmental reservoir of MBLs has been identified, which can act as a source for transfer into human pathogens. Therefore, structural investigation of environmental and clinically rare MBLs can give new insights into structure–activity relationships to explore the role of catalytic and second shell residues, which are under selective pressure.

**Objectives:**

To investigate the structure and activity of the environmental subclass B1 MBLs MYO-1, SHD-1 and ECV-1.

**Methods:**

The respective genes of these MBLs were cloned into vectors and expressed in *Escherichia coli*. Purified enzymes were characterized with respect to their catalytic efficiency (*k*_cat_/*K*_m_). The enzymatic activities and MICs were determined for a panel of different β-lactams, including penicillins, cephalosporins and carbapenems. Thermostability was measured and structures were solved using X-ray crystallography (MYO-1 and ECV-1) or generated by homology modelling (SHD-1).

**Results:**

Expression of the environmental MBLs in *E. coli* resulted in the characteristic MBL profile, not affecting aztreonam susceptibility and decreasing susceptibility to carbapenems, cephalosporins and penicillins. The purified enzymes showed variable catalytic activity in the order of <5% to ∼70% compared with the clinically widespread NDM-1. The thermostability of ECV-1 and SHD-1 was up to 8°C higher than that of MYO-1 and NDM-1. Using solved structures and molecular modelling, we identified differences in their second shell composition, possibly responsible for their relatively low hydrolytic activity.

**Conclusions:**

These results show the importance of environmental species acting as reservoirs for MBL-encoding genes.

## Introduction

The class B MBLs are enzymes with the ability to hydrolyse virtually all β-lactam antibiotics, including carbapenems.^1^ Various MBLs, including NDM, VIM and IMP, are associated with mobile genetic elements and widespread among clinically important Gram-negative pathogens. Phylogenetically, MBLs can be grouped into three subclasses, B1 to B3.^2^ While enzymes belonging to subclasses B1 and B3 carry two Zn(II) binding sites (Zn1 and Zn2), B2 MBLs are mono-Zn(II) enzymes.^2^^,^^3^ In subclass B1, Zn1 is coordinated by three histidine residues (His/Gly116, His118 and His196), while the Zn2 binding site is coordinated by Asp120, Cys221 and His263.^2^^,^^4–7^ In B2 MBLs, the Zn1 binding site displays one altered residue (Asn116, His118 and His196), whereas the Zn2 site is identical to that of the subclass B1 MBLs.^8^^,^^9^ The subclass B3 MBLs exhibit a variety of different Zn1 binding sites (His/Gln116, His118 and His196) and a distinct Zn2 binding site, which does not contain a cysteine residue (Asp120, His121 and His263). The Zn(II) ions are bridged by a hydroxide ion most likely attacking the β-lactam ring.^4^

Recently, 76 novel B1 MBL genes were predicted through large-scale screening of genomic and metagenomics data.^6^ Some of these enzymes exhibited sequence identities as low as 28% compared with widespread MBLs like NDM-1.^6^ Carbapenemase activity was experimentally confirmed for 18 of 21 tested MBLs when expressed in *Escherichia coli*.^6^^,^^10^ This shows that there is a vast environmental reservoir of MBL genes that could potentially be horizontally transferred into pathogenic bacteria and further compromise the effect of β-lactam antibiotics. Here, we investigated three of these B1 MBLs,^6^ SHD-1, MYO-1 and ECV-1, in comparison with the clinically widespread enzyme NDM-1. ECV-1 originated from *Echinicola vietnamensis*, which has previously been isolated from sea water.^11^ SHD-1 was identified in *Shewanella denitrificans*, a genus that is known as the possible origin of resistance genes, including genes encoding β-lactamases.^12^ MYO-1 was encoded on a *tet*(X)-harbouring plasmid in *Myroides odoratimimus*, a widely distributed bacterium in natural environments.^13–16^ The plasmid also encoded a type IV secretion system, which could make it conjugatable.^17^*M. odoratimimus* is not considered pathogenic under normal circumstances;^18^ however, it has been reported to cause opportunistic infections^19–22^ and treatment options are limited since most strains display MDR.^21^^,^^23–27^

## Methods

### Strains and MIC determination

All strains used for MIC determination have been published previously.^6^ In short, the candidate B1 MBL genes *bla*_MYO-1_, *bla*_ECV-1_ and *bla*_SHD-1_ were synthesized and sub-cloned into the pZE21-MSC1 vector (Expressys, Ruelzheim, Germany). Recombinant plasmids were transformed into *E. coli* C600Z1 (Expressys).^6^^,^^28^ For MIC determination, single colonies were incubated overnight on Mueller–Hinton II agar (Becton Dickinson, Franklin Lakes, USA) containing 25 mg/L kanamycin and subsequently suspended in 0.85% saline to a cell density with a turbidity equivalent to that of a 0.5 McFarland standard (1.5 × 10^7^ cells/mL). The McFarland solution was uniformly dispersed with a swab onto the agar plates containing 100 ng/mL anhydrotetracycline (Sigma–Aldrich, St Louis, MO, USA). Gradient diffusion strips (Liofilchem, Roseto degli Abruzzi, Italy) were applied and the MICs were determined after 19 h of incubation at 37°C.

### Enzyme expression, purification and molecular mass verification

For enzyme expression, we used synthetic and codon-optimized genes of *bla*_MYO-1_, *bla*_ECV-1_ and *bla*_SHD-1_ in a pDest17 vector (Thermo Fisher Scientific, Waltham, USA) with a TEV cleavage site placed prior to the *bla* genes. The genes were based on the *bla* genes found in *M. odoratimimus*,^23^*S. denitrificans* and *E. vietnamensis* (GenBank accession numbers CP013691.1, NC_007954.1 and NC_019904.1, respectively). The expression vectors were electroporated into *E. coli* BL21-AI (Invitrogen, Carlsbad, USA). For protein expression, cultures were induced with L-arabinose (0.1%; Sigma–Aldrich) at an OD_600_ of ∼0.5. Expression was performed in Terrific Broth including 100 mg/L ampicillin (Sigma–Aldrich) at 15°C and 225 rpm. TEV cleavage and purification were done as previously described.^29^ Due to the TEV cleavage site and expression without the signal peptide, the protein sequences start at position Gln30, Gly18, Val25 and Gly25 for MYO-1, ECV-1, SHD-1 and NDM-1, respectively (additional glycine at the start). NDM-1 was expressed and purified as described previously.^30^ For ESI-MS, the buffer was changed to 0.1% formic acid (Merck Millipore, Burlington, USA) in centrifugal molecular cut-off filters (Merck MilliPore, 10 000 Da) and concentrated to 0.25 g/L. The protein masses were verified using an Orbitrap Fusion Lumos (Thermo Fisher Scientific). Proteins were injected using an EASY-nano LC (Thermo Fisher Scientific) with a 15 cm C18 EASY-Spray column. Masses were calculated using the BioPharma Finder 3.0 protein deconvolution software (Thermo Fisher Scientific).

### Zn^66^ determination

Inductive coupled plasma MS (ICP-MS) was used to determine the Zn(II) concentration (Zn^66^) of purified protein in Zn(II)-depleted 50 mM HEPES buffer (Chelex-HEPES buffer), pH 7.5. The Chelex buffer was prepared by stirring 2 g of Chelex resin (Bio-Rad, Hercules, USA) in 100 mL of 50 mM HEPES buffer, pH 7.5. The resin was subsequently removed by sterile filtration (Merck MilliPore, 0.22 μm). Purified proteins (∼10 g/L) were diluted to 100 mg/L in Chelex-HEPES buffer. Residual Zn(II) was removed from the proteins by washing with Chelex-HEPES buffer in centrifugal molecular cut-off filters (Merck MilliPore, 10 000 Da). Samples were 1/16 diluted with 750 μL of a diluent mixture containing Rh103 (Inorganic Ventures, Christiansburg, VA, USA) as internal standard. The diluent mixture consisted of Milli-Q water (Millipore/Merck KGaA, Darmstadt, Germany) with 2 μg/L Rh103, 2.5% (v/v) ammonia solution (Honeywell Fluka, Bucharest, Romania), 0.08% (v/v) Triton X-100 (Sigma/Merck KGaA, Darmstadt, Germany), 10% (v/v) isopropanol (Honeywell Fluka) and 0.25 μg/L Au (Inorganic Ventures) as stabilizer. The samples were introduced to the nebulizer (N_2_ gas flow 1.03 mL/min) by an ESI-Fast SC2DX autosampler with a sample flow rate of 3 rpm and further into the NexION 300 D ICP-MS system (Perkin Elmer, Waltham, MA, USA). For the MS analysis the kinetic energy discrimination mode with a helium flow rate of 5.7 mL/min, 20 sweeps per reading and a dwell time of 100 ms/AMU for Zn(II) and 50 ms/AMU for Rh103 were applied. The measurements were performed with the following instrumental settings: rf power, 1600 W; plasma gas flow, 18 mL/min Ar; auxillary gas flow, 1.2 mL/min N_2_; RPQ voltage, 0.25 V; and integration time, 2000 ms. All Zn(II) concentrations were obtained by the internal standard method followed by a blank subtraction using the NexION software version 1.5 (Perkin Elmer, Waltham, MA, USA). The Zn(II) concentration within the samples was determined based on an external calibration curve.

### Thermostability

Fluorescence-based thermal stability of the enzymes was determined.^31^ In short, purified enzymes were diluted to 0.2 mg/mL using 50 mM HEPES buffer pH 7.5 supplemented with 100 μM ZnSO_4_ (Sigma–Aldrich) and 250 mM NaCl (VWR, Radnor, USA). For the fluorescence signal, 12.5× SYPRO orange (Sigma–Aldrich) was used. Melting curves were recorded across a temperature gradient (10–75°C). Tests were performed in an MJ Minicycler (Bio-Rad, Hercules, USA) and melting temperatures were calculated by using the Bio-Rad CFX Manager (v. 3.1). All experiments were carried out in a final volume of 25 μL and at least in triplicate. Purified NDM-1 was included as a control.

### Steady-state enzyme kinetics

*K*_m_ and *k*_cat_ for recombinantly expressed enzymes were determined for ampicillin (Δξ = −820 M^−1 ^cm^−1^, 235 nm, 1 nM), piperacillin (Δξ = −820 M^−1 ^cm^−1^, 235 nm, 1 nM), nitrocefin (Δξ = 17 400 M^−1 ^cm^−1^, 482 nm, 1 nM), ceftazidime (Δξ = −9000 M^−1 ^cm^−1^, 260 nm, 150 nM), cefepime (Δξ = −10 000 M^−1 ^cm^−1^, 260 nm, 1 nM), imipenem (Δξ = −9000 M^−1 ^cm^−1^, 300 nm, 1 nM) and meropenem (Δξ = −6500 M^−1 ^cm^−1^, 300 nm, 1 nM) by measuring the initial enzymatic reaction rate at 25°C. All determinations were performed at least in duplicate at a final assay volume of 100 μL. For nitrocefin-dependent reactions, 96-well plates (Thermo Fisher Scientific, Roskilde, Denmark) were utilized. For all the other drugs, UV-transparent 96-well plates (Corning, Kennebunk, ME, USA) were used. All tests were performed in HEPES buffer 50 mM supplemented with 10 μM ZnSO_4_ (Sigma–Aldrich) and BSA (Sigma–Aldrich) at a final concentration of 2 μg/mL. Calculations were performed by using GraphPad Prism^®^ 7.0 (GraphPad Software Inc., USA).

### Crystallization and structure determination

For ECV-1 (5 mg/mL), crystals were grown from reservoirs with 25%–26% PEG3350 (Sigma–Aldrich), 0.1 M BIS-TRIS buffer pH 6 (Sigma–Aldrich) and 0.2 M sodium acetate (Sigma–Aldrich) at 4°C. Crystal-containing drops were diluted with 10 μL of reservoir solution and microcrystals were created. Microcrystals were seeded into drops of 2 μL containing the same composition and 5 mg/mL purified protein. For MYO-1 (5 mg/mL), crystals were grown in 32%–36% PEG4000 (Sigma–Aldrich) and 0.2 M ammonium sulphate at 4°C (drop size 2 μL). Crystals were flash-frozen in liquid nitrogen using 10% ethylene glycol (Sigma–Aldrich) in addition to the reservoir solution. Since crystallization of SHD-1 was not successful, we used SWISS-MODEL and the solved structure of TMB-1 (PDB ID: 5MMD) with sequence identity of 58%, to obtain a homology-modelled structure.^29^^,^^32^

Diffraction data were collected at ID30A-3, at the European Synchrotron Radiation Facility (ESRF), France, at 100 K, wavelength of 0.961 Å, and the diffraction images were indexed and integrated using XDS.^33^ AIMLESS was used for scaling.^34^ For scaling, we aimed for high completeness, a CC_1/2_ >0.5 in the outer resolution shell and a mean <I> above 1.0 (Table 1). Both structures were solved by molecular replacement using PDB ID: 1ZNB (ECV-1) and 1HLK (MYO-1) as search models and refined using Phenix 1.12.^35^ Modelling was done using Coot.^36^ Figures were prepared using PyMOL version 1.8 (Schrödinger).

**Table 1.** X-ray data collection and refinement statistics^a^

	MYO-1	ECV-1
Data collection	ESRF, ID30A-3	ESRF, ID30A-3
PDB entry	6T5L	6T5K
wavelength (Å)	0.961	0.961
space group	P6_5_	C222_1_
cell dimensions: a, b, c (Å)	144.68, 144.68, 53.31	51.93, 65.68, 128.50
resolution (Å)	25.0–2.17 (2.25–2.17)	24.08–1.33 (1.38–1.33)
*R*_merge_	0.041 (0.790)	0.042 (0.591)
I/σI	11.2 (1.1)	8.1 (1.1)
completeness (%)	98.8 (99.6)	99.5 (95.4)
redundancy	3.5 (3.6)	5.6 (3.9)
CC_1/2_	0.998 (0.413)	0.999 (0.489)
Refinement		
resolution (Å)	25.0–2.17	24.08–1.33
no. reflections	33 586	49 518
*R*_work_/*R*_free_	0.2172/0.2526	0.1550/0.1879
no. H atoms	3605	1794
protein	3458	1764
ligand/ion	5	83^d^
water	142	254
B factors (Å^2^) on average	52.4^b^/85.2^c^	24.9
protein	52.3^b^/85.7^c^	23.4
ligand/ion	49.8^b^/75.2^c^	34.4
water	54.8^b^/57.6^c^	34.9
r.m.s. deviations		
bond lengths (Å)	0.014	0.019
bond angles (°)	1.31	1.50

a Values in parentheses are for the highest-resolution shell.

b B factors of MYO-1 chain A.

c B factors of MYO-1 chain B.

d Including five molecules of ethylene glycol.

## Results

### Environmental MBLs decrease susceptibility to β-lactams in E. coli

**Figure 1.**
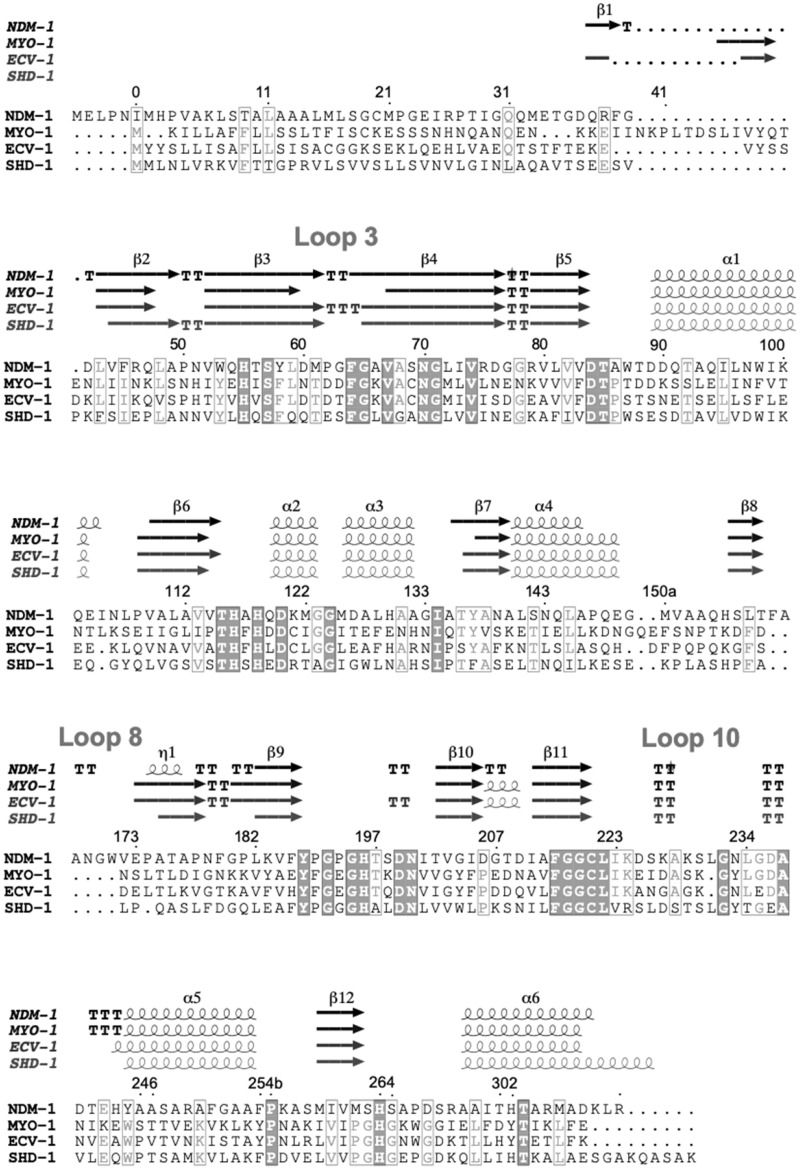
Multiple sequence alignment based on the MBL numbering system.^72^ For calculating the secondary structure elements, we used the published structure of NDM-1 (PDB ID: 3ZR9).^40^ Sequence identity compared with NDM-1 was determined for MYO-1 (28%), ECV-1 (33%) and SHD-1 (33%). The alignment shows conserved (filled boxes) and semi-conserved (grey font) residues within the selection.^72^ TT and TTT indicate β-turns and α-turns, respectively.

The sequence identity of MYO-1, ECV-1 and SHD-1 was as low as 28% compared with the widespread MBL NDM-1 (Figure 1). We identified differences in their loop regions L3 (residues 56–66), L8 (residues 151–160) and L10 (residues 220–237), which are involved in Zn(II) binding and defining substrate specificity.^4^ In addition, MYO-1 and ECV-1 harboured in total three cysteine residues (positions 69, 121 and 221) within their active site. To explore if the differences in the amino acid sequence could potentially influence the substrate specificity, we performed susceptibility testing of *E. coli* expressing MYO-1, ECV-1 and SHD-1. The respective genes (not codon-optimized) were sub-cloned into pZE21-MSC1 and expression was induced with anhydrotetracycline in *E. coli* C600Z1 (Table 2). NDM-1 was included for comparison. All three enzymes showed the characteristic MBL profile, increasing the MIC of all β-lactams except for aztreonam. MBL activity was also confirmed by inhibition with EDTA. SHD-1 conferred the highest increase in carbapenem MICs, with a 64-, 4- and 1024-fold increase for ertapenem, imipenem and meropenem, respectively (compared with *E. coli* C600Z1). The observed effect on carbapenem MICs was lower for MYO-1 and ECV-1. Still, the expression of MYO-1 and ECV-1 resulted in an 8- and 16-fold increase in their ertapenem MICs and a 16- and 8-fold increase in their meropenem MICs, respectively. In addition, MYO-1 led to a 4-fold increase in the imipenem MIC. Compared with NDM-1, which conferred MIC values of cephalosporins of up to >256 mg/L, the MICs of cephalosporins tended to be lower for all the environmental MBLs, ranging from 0.25 to >256 mg/L (4- to >512-fold change depending on the cephalosporin). With the exception of piperacillin for MYO-1 and ECV-1, the MICs of penicillins were increased by >4- to >32-fold. For MYO-1 and ECV-1, an 8- and 4-fold increase in their MICs of piperacillin was observed compared with a >256-fold increase for NDM-1 and SHD-1, respectively.

**Table 2.** MICs (mg/L) for *E. coli* C600Z1 expressing *bla*_MYO-1_, *bla*_ECV-1_ and *bla*_SHD-1_ sub-cloned into the pZE21-MSC1 expression vector; *bla*_NDM-1_ was included as a comparator and empty vector was included as a control

	*E. coli* C600Z1	*E. coli* C600Z1 pZE21-MSC1	*E. coli* C600Z1 pZE21-MSC1			
			*bla*_MYO-1_	*bla*_ECV-1_	*bla*_SHD-1_	*bla*_NDM-1_
Penicillins						
ampicillin	8	8	>256	>256	>256	>256
penicillin G	64	64	>256	>256	>256	>256
piperacillin	2	1	16	8	>256	>256
Cephalosporins						
cefepime	0.064	0.064	8	0.25	2	>256
cefotaxime	0.5	1	16	8	>32	>32
cefoxitin	8	12	64	128	>256	>256
ceftazidime	0.5	0.25	>256	16	>256	>256
cefalotin	32	32	>256	>256	>256	>256
Carbapenems						
ertapenem	0.032	0.032	0.25	0.5	2	16
imipenem	0.25	0.25	1	0.25	1	>32
meropenem	0.032	0.064	0.5	0.25	32	>32
meropenem/EDTA	<0.032	<0.032	<0.032	<0.032	<0.032	<0.032
Monobactam						
aztreonam	0.25	0.25	0.25	0.25	0.25	0.25

### Environmental β-lactamases possess lower activity than NDM-1

Synthetic, codon-optimized genes were used to overexpress MYO-1, ECV-1 and SHD-1 in *E. coli*. Protein purification yielded 50 mg (MYO-1), 9 mg (ECV-1) and 62 mg (SHD-1) per litre of culture. The purity of the enzymes was >95%. Computed monoisotopic mass of tag-free MYO-1, ECV-1 and SHD-1 was confirmed by ESI-MS to be 26 771.6 ± 3.3, 26 348.2 ± 0.3 and 25 741.2 ± 1.1 Da, respectively. The Zn(II) content of MYO-1, ECV-1, SHD-1 and NDM-1 was determined by ICP-MS and we found 2.0 ± 0.1, 1.9 ± 0.1, 1.7 ± 0.1 and 1.7 ± 0.1 Zn(II) atoms per enzyme, respectively. Thermostability measurements resulted in melting temperatures of 57.8 ± 0.1, 60.8 ± 0.3, 66.2 ± 0.4 and 57.9 ± 0.1°C, respectively.

**Figure 2.**
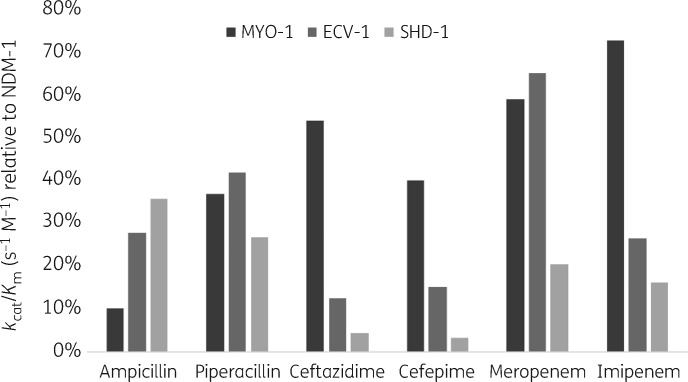
Relative catalytic efficiencies [*k*_cat_/*K*_m_ (s^−1^ M^−1^)] of MYO-1, ECV-1 and SHD-1 compared with NDM-1.

All enzymes showed catalytic activity against the tested β-lactams (Table 3). In general, SHD-1 showed the lowest enzymatic activity. Against penicillins and carbapenems, the catalytic activity of SHD-1 was usually 2- to 4-fold lower compared with MYO-1 and ECV-1. The activities of MYO-1 and ECV-1 were generally comparable to each other. The cefepimase and ceftazidimase activity of MYO-1 was ∼10-fold higher than that of SHD-1. The lower activity of SHD-1 towards cephalosporins was due to both lower affinity (*K*_m_ >300 μM) and lower turnover (*k*_cat_ ≤10 s^−1^). In line with the MIC results, the β-lactamase activities of the environmental MBLs were lower, ranging from <5% to ∼70%, compared with NDM-1 (Figure 2). For MYO-1, the catalytic activity tended to be higher and its carbapenemase activity reached up to ∼70% to that of NDM-1. On the contrary, SHD-1 displayed the weakest comparative carbapenemase and cephalosporinase activity, with values generally below 10%. In addition, ECV-1 demonstrated high catalytic activity towards meropenem (65% compared with NDM-1), whereas imipenem, penicillins and cephalosporins were hydrolysed to a lower degree (∼10%–40%).

**Table 3.** Kinetic values (*k*_cat_, *K*_m_ and *k*_cat_/*K*_m_) of recombinantly expressed and purified MYO-1, ECV-1, SHD-1 and NDM-1; errors are reported as standard errors

Substrate	MYO-1			ECV-1			SHD-1			NDM-1		
	*k*_cat_ (s^−1^)	*K*_m_ (μM)	*k*_cat_/*K*_m_ (s^−1^ M^−1^)	*k*_cat_ (s^−1^)	*K*_m_ (μM)	*k*_cat_/*K*_m_ (s^−1^ M^−1^)	*k*_cat_ (s^−1^)	*K*_m_ (μM)	*k*_cat_/*K*_m_ (s^−1^ M^−1^)	*k*_cat_ (s^−1^)	*K*_m_ (μM)	*k*_cat_/*K*_m_ (s^−1^ M^−1^)
Ampicillin	130 ± 9	1200 ± 180	1.1 × 10^5^	100 ± 10	340 ± 100	2.9 × 10^5^	70 ± 3	180 ± 16	3.9 × 10^5^	70 ± 4	60 ± 13	1.1 × 10^6^
Piperacillin	70 ± 3	140 ± 20	5.0 × 10^5^	300 ± 20	550 ± 90	5.5 × 10^5^	70 ± 10	180 ± 52	3.9 × 10^5^	180 ± 17	140 ± 55	1.3 × 10^6^
Cefepime	30 ± 3	180 ± 40	1.7 × 10^5^	5 ± 1	70 ± 11	7.1 × 10^4^	8 ± 2	550 ± 160	1.5 × 10^4^	12 ± 4	30 ± 4	4.0 × 10^5^
Ceftazidime	50 ± 4	130 ± 30	3.9 × 10^5^	30 ± 3	340 ± 57	8.8 × 10^4^	10 ± 1	340 ± 72	2.9 × 10^4^	12 ± 1	20 ± 4	6.0 × 10^5^
Imipenem	40 ± 1	50 ± 6	8.0 × 10^5^	20 ± 1	60 ± 6	3.3 × 10^5^	40 ± 2	210 ± 20	1.9 × 10^5^	8 ± 1	75 ± 4	1.1 × 10^6^
Meropenem	20 ± 1	40 ± 6	5.0 × 10^5^	50 ± 3	70 ± 10	7.1 × 10^5^	8 ± 1	40 ± 4	2.0 × 10^5^	50 ± 1	45 ± 3	1.1 × 10^6^

### First shell, second shell and substrate binding residues

**Figure 3.**
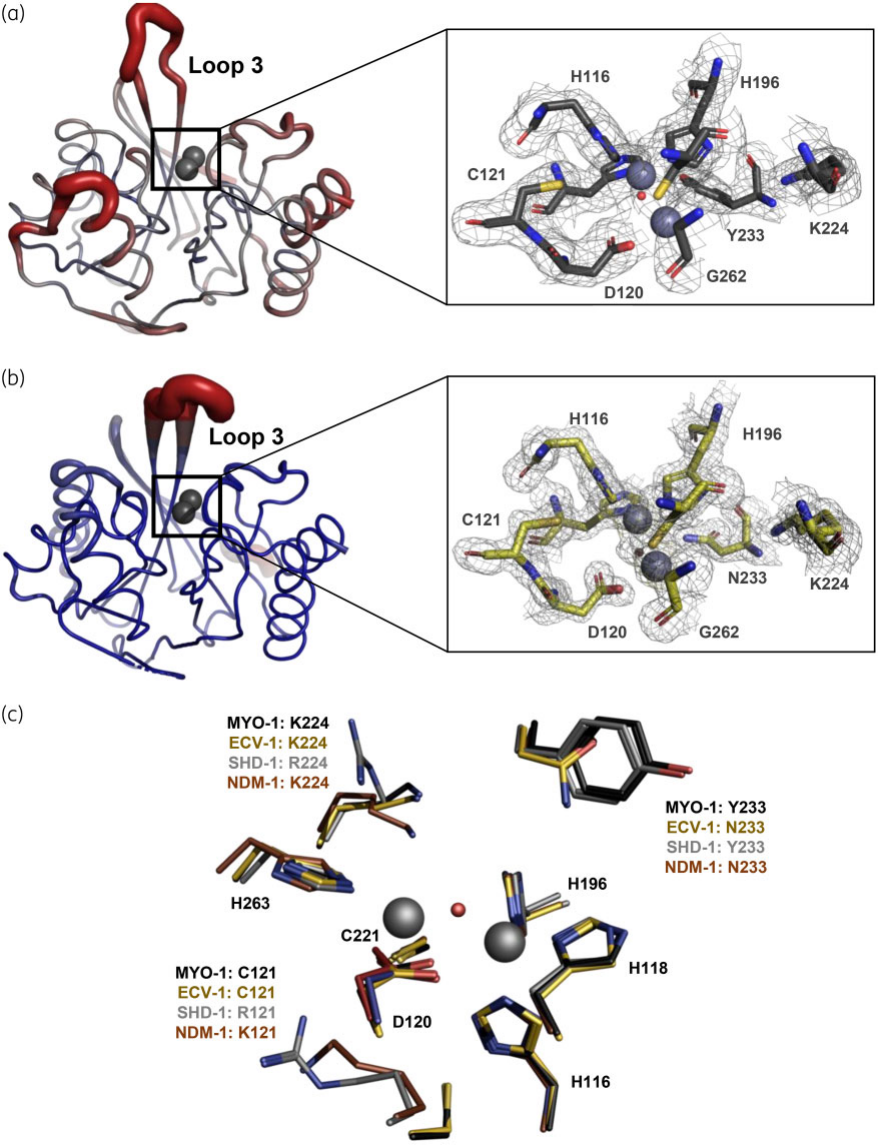
Overall fold of (a) MYO-1 (chain A) and (b) ECV-1 with the crystallographically assigned B values (left), where blue represents low B factors and red represents high B factors (colour code scaling of B factors from 20 to 75 Å^2^), and their active site amino acids including the corresponding 2Fo-Fc map (right). Temperature factors for MYO-1 were generally higher than for ECV-1. However, both structures showed high variation in their loop 3 region. (c) Active site of MYO-1 (black), ECV-1 (gold) and SHD-1 (grey) superimposed onto NDM-1. The Zn(II) ions are displayed from MYO-1.

The structures of MYO-1 and ECV-1 were successfully solved by X-ray crystallography to 2.17 and 1.33 Å, respectively (Figure 3a and b and Table 1). For MYO-1 we found two molecules (chains A and B) in the asymmetrical unit with *R*_work_ and *R*_free_ of 0.22 and 0.25 (space group P6_5_). Due to lack of electron density in chain B, the regions of N60 to K66, L93 to I96 and K104 to S105 could not be built. The structure of ECV-1 was refined to an *R*_work_ and *R*_free_ of 0.16 and 0.19, respectively, with one molecule in the asymmetrical unit. For SHD-1, we used homology modelling since no crystal structure was obtained. In addition, we found that the conserved active site residues (first shell) coordinating Zn1 (H116, H118 and H196) and Zn2 (D120, C221 and H263) were present in all three enzymes (Figure 3c).

**Figure 4.**
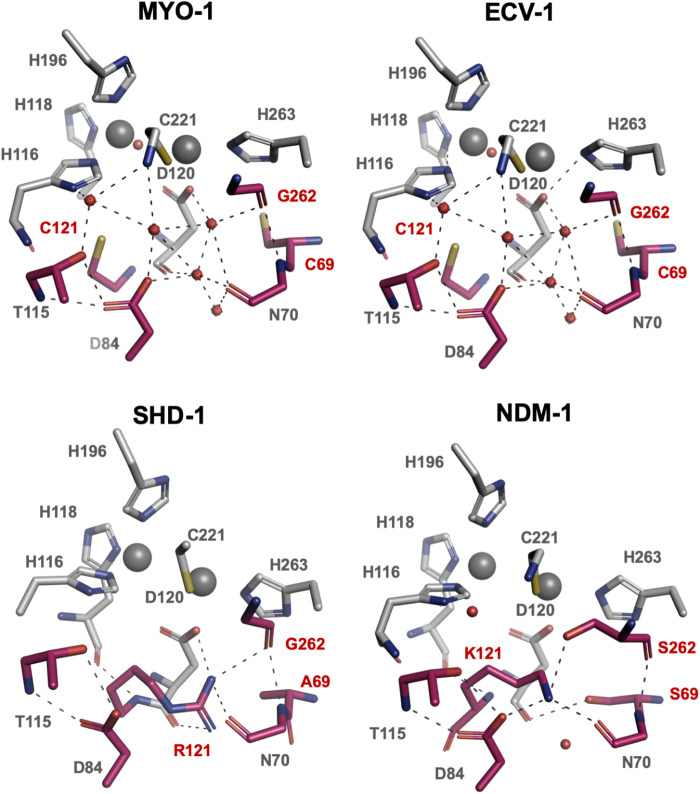
First and second shell residues of MYO-1, ECV-1, SHD-1 and NDM-1. First shell residues are displayed in grey and second shell residues are shown in red. Labels of amino acids varying between these four enzymes at positions 69, 121 and 262 are displayed in red. The lack of K121 or R121 in MYO-1 and ECV-1 is compensated for by water molecules.

B1 MBLs usually share an H-bond network below the active site involving second shell residues.^37–39^ These residues have been shown to modulate substrate specificity and Zn(II) binding.^39^ Generally, the residues at positions 69, 70, 84, 115, 121 and 262 are part of this H-bond network (Figure 4). To investigate this H-bond network, we superimposed the structures of MYO-1, ECV-1 and SHD-1 onto NDM-1 (PDB ID: 3ZR9).^40^ Superimposition resulted in low root mean square deviation of 0.97, 1.03 and 0.92 Å, respectively, and 0.72 Å for MYO-1 versus ECV-1. In contrast to the complex H-bond network in NDM-1 involving Ser69, Asp84, Lys121 and Ser262, we found different amino acids in MYO-1 (Cys69, Cys121, Gly262), ECV-1 (Cys69, Cys121, Gly262) and SHD-1 (Ala69, Arg121, Gly262) (Figure 4). Asp84 was conserved in all four enzymes. Compared with Arg121 (SHD-1) and Lys121 (NDM-1), we found a third cysteine (Cys121) within the active site of MYO-1 and ECV-1. These cysteines (Cys69, Cys121, Cys221) were in the vicinity of Asp120 (3–5 Å). The lack of Lys121 or Arg121 in MYO-1 and ECV-1 was compensated for by an extensive network of water molecules (Figure 4). The L10 loop (residues 220–237) has been described to be involved in Zn(II) binding and substrate specificity, where the interaction with the substrate was due to hydrophobic contacts.^37^ Interestingly, this loop was shortened by one residue at position 231 in both MYO-1 and ECV-1. Moreover, we found variation in residues at positions 224 and 233, which have been reported to play an important role in substrate recognition and hydrolysis in NDM and VIM variants.^41^^,^^42^ At position 233, asparagine was present in both NDM-1 and ECV-1; however, we found tyrosine in MYO-1 and SHD-1. In addition, we identified the amino acid substitution K224R in SHD-1 compared with NDM-1, ECV-1 and MYO-1.

## Discussion

Here, we present two new crystal structures and one homology model of MBLs identified in environmental bacteria.^6^ Expressed in *E. coli*, all three enzymes conferred decreased susceptibility to carbapenems, cephalosporins and penicillins. Compared with NDM-1, the expression of MYO-1, ECV-1 and SHD-1 led to lower MICs, especially those of carbapenems (Table 2). We determined the catalytic efficiency using purified enzymes. Generally, the enzymatic activity ranked MYO-1 > ECV-1 > SHD-1. We found the largest differences in catalytic efficiency towards cephalosporins, where MYO-1 exhibited up to 44-fold higher activity against cefepime compared with SHD-1. Interestingly, SHD-1 conferred the highest MIC values when expressed in *E. coli*, but the lowest catalytic efficiencies (purified enzyme). SHD-1 was identified in a Gammaproteobacterium, while the natural hosts of MYO-1 and ECV-1 belong to the distant phylum of Bacteroidetes^6^ and hence may not be expressed efficiently in the periplasm of *E. coli*. Work on the subclass B1 SPM-1 has shown different drug selectivity when tested in the periplasm, in enzyme kinetic assays and in an MIC set-up.^39^ In addition, the expression of the same class B and D β-lactamases in different hosts exhibited a lack of correlation between MICs and the catalytic efficiency of these enzymes.^43^^,^^44^ Hence, phenotypic variation can be due to differences in catalytic efficiency in the periplasmic conditions, but expression level, protein folding and translocation to the periplasm can also play a role.^43^

ECV-1 and SHD-1 exhibited thermostabilities ∼3 and ∼8°C higher than MYO-1 and NDM-1. Studies have shown that lower thermostability was accompanied by higher flexibility, facilitating cephalosporin hydrolysis in β-lactamases.^45^^,^^46^ Interestingly, the more thermostable SHD-1 and ECV-1 showed lower catalytic efficiency, especially against oxyimino cephalosporins. However, due to the low sequence identity (∼28%) further studies have to be conducted exploring the structure–activity relationships and a possible correlation with thermostability.

Since second shell residues have been reported to be under evolutionary pressure and their substitutions have created variants with changed enzymatic activity,^38^^,^^47^^,^^48^ we investigated the structures of MYO-1, ECV-1 and SHD-1. We found the positions 69, 121 and 262 differed from the second shell residues of NDM-1. In NDM-1, mutational studies of Ser69 and Lys121 revealed that a cysteine replacement, as present in MYO-1 and ECV-1, reduced bacterial fitness towards cefotaxime and imipenem, while Ala69 and Arg121 (SHD-1) improved bacterial survival after selection.^49^ The amino acid position 121 is semi-conserved as it is directly situated ‘below’ the Zn2 binding site. While crystallographic occupancy correlated with reduced Zn(II) affinity for MBLs carrying Arg121 (e.g. BcII, VIM-2 and BlaB),^50^^,^^51^ high occupancy was seen for MBLs carrying serine or cysteine at this position, e.g. IMP-1 and CcrA.^52–54^ Mutational studies of BcII:R121C showed a marginal increase in occupancy compared with WT BcII.^55^ In contrast, C121R in CcrA resulted in a variant with lower Zn(II) affinity.^56^ Arg121 interacts with Asp120 in BcII and data suggest that R121C may affect the p*K*_a_ of Asp120, thus changing the pH-dependent activity of the enzyme.^57^ Interestingly, in BcII:R121C a network of water molecules populates the active site as a replacement for a guanidinium group of arginine that usually preserves its shape.^55^ G262S differentiates IMP-1 from IMP-6 and has been shown to also enhance catalytic efficiency in both IMP and BcII.^58^ Precursor enzymes of IMP-1 have therefore been reported to be less active against, for example, ampicillin, ceftazidime and imipenem.^59^^,^^60^ In addition, an amino acid substitution of G262S in IMP-1 suggested reduced mobility of His263 by the formation of an H-bond network allowing the accommodation of cephalosporins.^61–63^ We confirmed the presence of an extensive water-mediated H-bond network within the active site of MYO-1 and ECV-1, likely to be caused by the presence of Cys121 and Gly262 (Figure 4). Tyr244 in NDM-1 has been shown to stabilize the L10 loop by the formation of hydrophobic interactions with, for example, Leu222, Leu231 and Leu234.^64–69^ We found Leu231 to be deleted in MYO-1 and ECV-1 as well as a substitution at position 234 to threonine in SHD-1. Mutational studies of NDM-1 with L231F resulted in a decreased hydrolytic activity towards carbapenems, penicillins and cephalosporins.^70^ The amino acids located at 224 and 233 have been reported to be important in substrate recognition and hydrolysis.^41^^,^^42^

In conclusion, this work presents the structure and activity of three MBLs from environmental sources. We showed that these enzymes act as carbapenemases exhibiting increased catalytic activity and conferring elevated MICs when expressed in *E. coli*. The lower activity towards cephalosporins and carbapenems could be, at least partially, explained by their second shell residues. These residues have been previously shown to be under selective pressure in other enzymes, and amino acid substituents may alter Zn(II) binding and extend their substrate specificity.^38^^,^^47^^,^^48^^,^^56–60^ Mobilization and horizontal transfer of genes expressing these or similar enzymes into clinical strains may render those strains less susceptible towards carbapenems and carbapenemase inhibitors acting as Zn(II) chelators.^71^

## Funding

This work was funded by the Swedish Research Council (2013-08633 and 2018-02835).

## Transparency declarations

None to declare.
